# Unusual segmental ischemia of the small bowel from cocaine abuse

**DOI:** 10.1093/jscr/rjab074

**Published:** 2021-04-14

**Authors:** Alfonso Grottesi, Leonello Bianchi, Francesco Maria Ranieri, Ernesto Puce, Marco Catarci

**Affiliations:** 1 General Surgery Unit, Sandro Pertini Hospital – ASL Roma 2, Roma, Italy; 2 Anesthesia and Intensive Care Unit, Sandro Pertini Hospital – ASL Roma 2, Roma, Italy; 3 Emergency Medicine Unit, Sandro Pertini Hospital, ASL Roma 2, Roma, Italy

## Abstract

Cocaine abuse is rising in the young population, triggering uncommon and potentially life-threatening causes of acute abdomen in this age group. The authors present the case of a 30-year-old man with emergency admission due to abdominal pain, with no history of drug abuse. Several signs and symptoms elicited toxicologic blood screening, which disclosed high serum levels of cocaine and its metabolites. Twelve hours after admission, the onset of acute abdomen with signs of diffuse peritonitis prompted surgical exploration through a minimally invasive approach. Two segmental small bowel ischemic loops and diffuse peritonitis, but no bowel perforation, were identified and treated by laparoscopic peritoneal lavage with 5 l of heated saline and intravenous administration of sodium heparin, 10 000 IU. Postoperative course was uneventful with home discharge on postoperative day 5. High index of suspicion is required to establish a prompt diagnosis and treatment of this uncommon cocaine abuse-related disease.

## INTRODUCTION

Cocaine abuse rose exponentially among the Italian population during the last 10 years; it is estimated to affect 5–10% of young people aged 16–30 years [1]. This substance abuse triggered the appearance of unusual clinical scenarios in this age range, with segmental ischemia of the small bowel being one of the less common [2]. The clinical presentation of small bowel ischemia in the young adult is very variable and, together with reluctance to admit drug abuse, it can lead clinicians to misdiagnosis and/or diagnostic delay with serious consequences for the patient [3].

## CASE REPORT

A 30-year-old male patient was admitted to the emergency room with 6-h history of headache, diffuse abdominal pain and fever (>38°C), diarrhea and vomiting. Previous medical history disclosed no alcohol or drugs assumption, no chronic disease. On arrival: fair general conditions, Glasgow Coma Scale (GCS): 15, blood pressure: 170/90, rhythmic heart rate at 72 bpm, body temperature: 36.8°C, SO_2_ 100% in room air and cardiovascular, nervous and abdominal examinations: normal. An electrocardiogram disclosed the presence of sharp T waves. Normal findings were found at head computed tomography (CT) scan and at abdominal plain X-rays. Laboratory data were normal, apart from a slight neutrophilia (76.2%; nv: 37–75%), mild sodium deficiency (134 mEq/l; nv: 137–145) and a moderate increase in blood glucose (184 mg/dl; nv: 74–106). Serum toxicology tests showed high values of cocaine and its metabolites, and the patient admitted its chronic abuse. Flaring up of abdominal pain prompted abdominal  ultrasound  (US)  scan, revealing an abnormal diffuse

**Figure 1:**
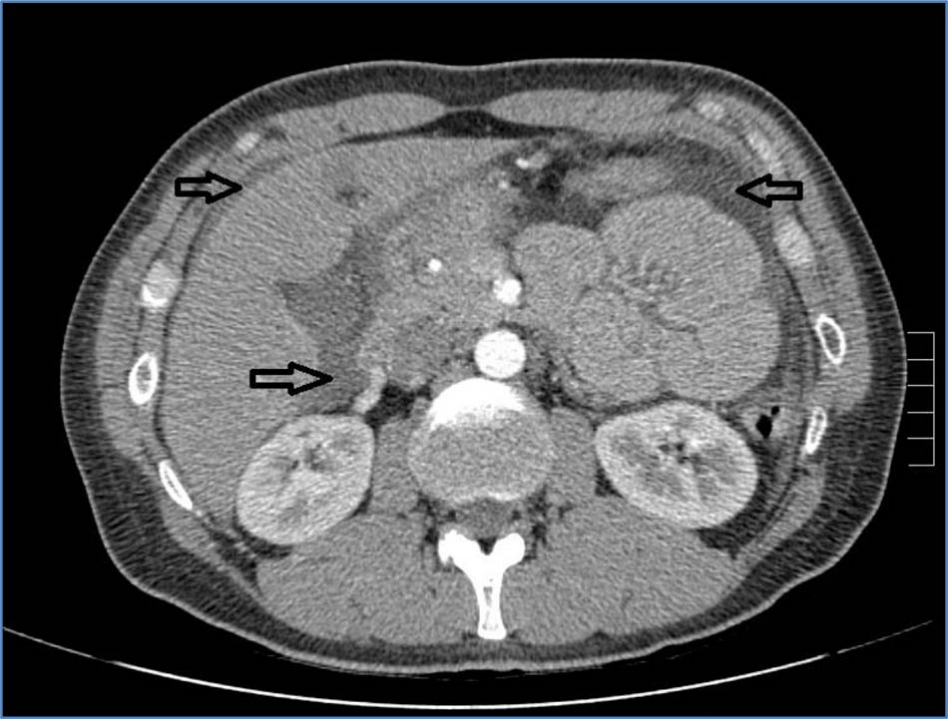
Abdominal CT scan showing diffuse fluid collection (arrows).

**Figure 2:**
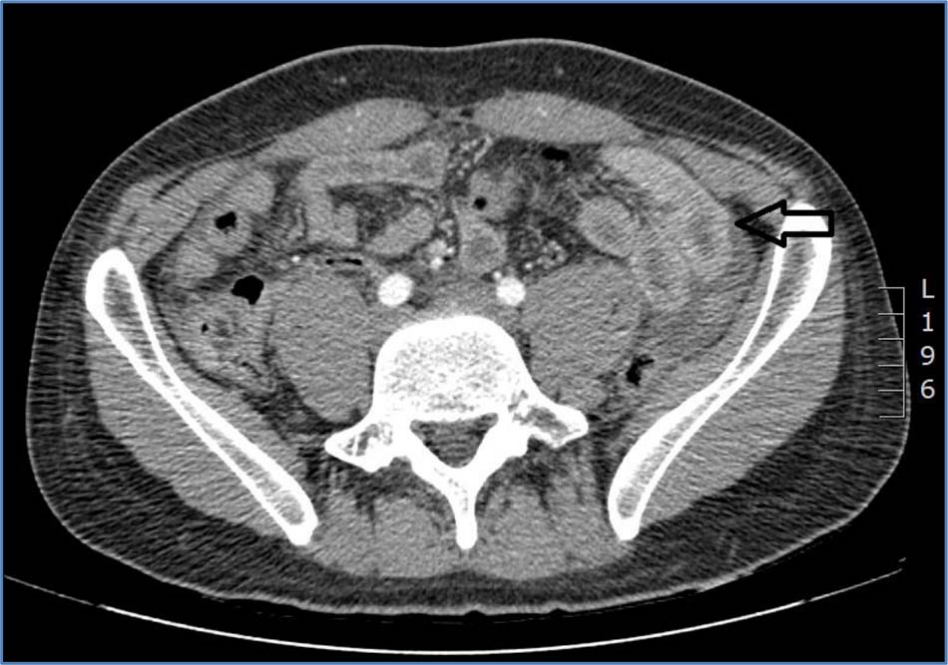
Abdominal CT scan showing distention, bundling and thickening of some small bowel loops in the left quadrant (arrow).

fluid collection in the peritoneal cavity. Subsequent CT confirmed the presence of diffuse fluid collection in the peritoneal cavity (Fig. 1) but no free air, with distention, bundling and thickening of some small bowel in the left quadrant (Fig. 2). The patient was therefore admitted for supporting therapy (intravenous fluids and antibiotics) with the suspicion of cocaine-induced small bowel ischemia. Twelve hours later, signs and symptoms of diffuse peritonitis developed: worsening diffuse abdominal pain with rebound guarding at examination, a significant increase in WBCs count (16.15 × 10^3^/μl) and neutrophilia (88%), lengthening of coagulation times (INR: 1.68; aPTT: 33.0 s) and increase in fibrinogen (430 mg/dl) and D-Dimer (2275 ng/ml). Therefore, urgent surgical exploration of the abdomen through a three-port open laparoscopic approach was performed, confirming the presence of 1200 ml of exudative fluid and fibrin clots (Fig. 3). Several adhesions between greater omentum, abdominal wall and small bowel underwent blunt dissection. Thorough exploration of the small bowel revealed two ischemic segments located at about 50 and 100 cm from the Treitz ligament (Fig. 4). The large bowel appeared normal. The existence of non-visible gastro-duodenal perforations was ruled out with the administration of 500 ml of saline with 20 ml of methylene blue through the nasogastric tube. An iv bolus of sodium heparin, 10 000 IU, was then administered, performing a 30-min peritoneal lavage with 5 l of saline solution at 37°C. After reaspiration of peritoneal lavage, a new complete exploration of the small bowel was performed, showing complete revascularization of the two ischemic small bowel segments (Fig. 5). Two tubular drainage tubes were placed in the left and right colic gutters. Culture examination of peritoneal exudate was positive for multi-sensitive *Escherichia coli* and *Enterococcus*, thus confirming bacterial translocation peritonitis. Postoperative therapy included the administration of iv piperacillin–tazobactam and subcutaneous low molecular weight heparin. Postoperative recovery was smooth, with oral feeding on the second postoperative day (POD), drainage removal on POD 3, complete bowel function restored on POD 4 and home discharge on POD 5. At 15- and 30-day-follow-up, the patient showed full recovery, being now followed by a specialized support structure for his drug abuse.

**Figure 3:**
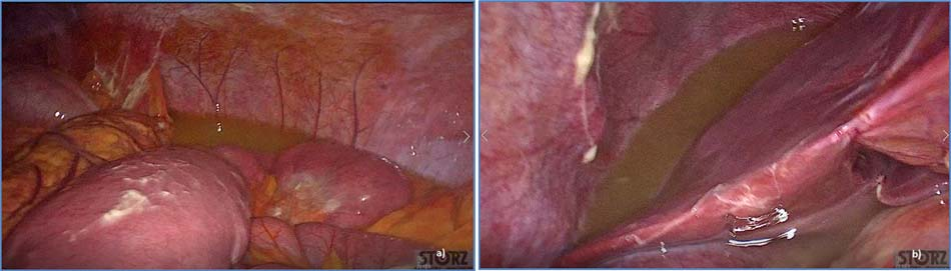
Diffuse exudative fluid and fibrin clots at laparoscopic exploration: (**a**) left upper quadrant and (**b**) right upper quadrant.

**Figure 4:**
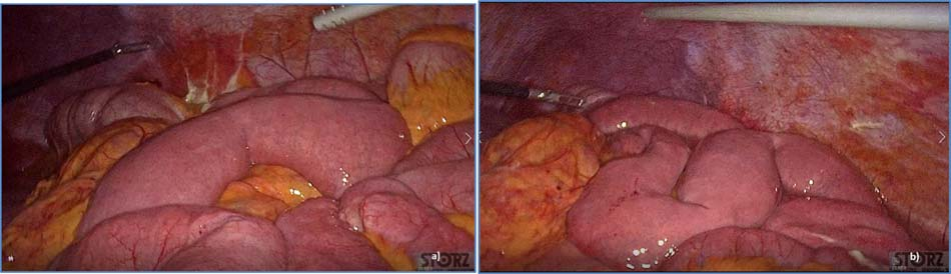
Dilated small bowel loops at laparoscopic exploration: bowel ischemia is evident from the absence of visible vasa recta compared to the adjacent loops (**a**) 50 cm and (**b**) 100 cm from the ligament of Treitz.

**Figure 5:**
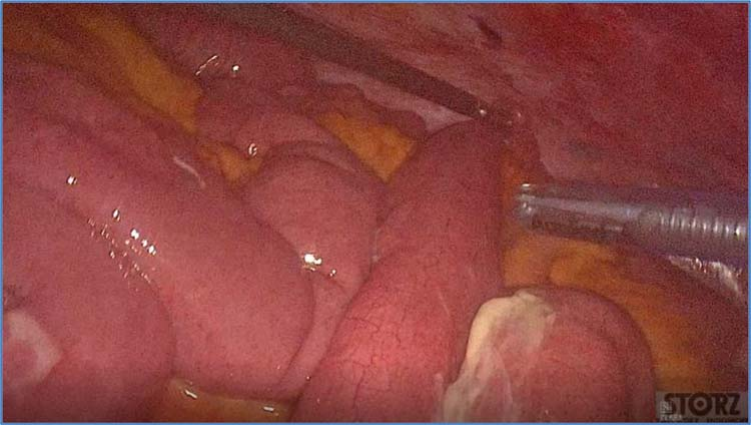
Progressive small bowel revascularization perceived by slow reappearance of vasa recta after peritoneal lavage.

## DISCUSSION

The vasoactive effects of cocaine and metabolites are relatively easy to detect. These affect the cardiac and cerebrovascular systems, with serious consequences on the morbidity and mortality of patients with these substances abuse [4, 5]. Much less common are the effects observed on the gastrointestinal system: among them, ischemic ulcers of the stomach and duodenum [6] or segmental colitis or pancolitis [7] are well known, while those affecting the small bowel are rather unusual [3]. The diffuse awareness that cocaine abuse in subjects aged 50 years or more significantly increases the incidence of myocardial or cerebral ischemia, gastroduodenal ulcers and ischemic colitis generally allows a prompt, possibly conservative, treatment, thus reducing organ damage. On the other hand, small bowel ischemia, quite rare among younger people, together with the high reluctance of younger patients to admit cocaine abuse, may pose definite problems concerning its differential diagnosis. In the present case, only the high index of suspicion by the emergency room doctor led to carry out a blood test to check for any trace of substances abuse, making it possible to consider the proper diagnosis prior to the onset of an acute abdomen triggered by cocaine-induced bowel ischemia. Cocaine abuse can cause mesenteric ischemia and gangrene, which result in small and large bowel perforation as well as intra-peritoneal hemorrhage [8]. The distal ileum is most commonly affected, but there are reports of gangrene involving almost any part of the small bowel. The common underlying pathophysiological mechanism is cocaine-induced arterial vasospasm or vasoconstriction leading to intestinal ischemia with mucosal and transmural necrosis [9]. Although successful conservative treatment has been reported, most patients with small bowel ischemia usually require urgent surgical exploration for peritonitis [10]. The rise of cocaine abuse in the young population entails a very important diagnostic problem regarding uncommon diseases in this age group, such as acute small bowel ischemia; in most cases, the related morbidity and mortality are time-dependent, and a high index of suspicion is therefore required to establish a prompt and proper diagnosis; this can allow a minimally invasive surgical treatment without bowel resection as in the present case, limiting serious consequences for the quality of life and mortality of patients.
